# Primary ovarian insufficiency in adolescents: a case series

**DOI:** 10.1186/s13633-015-0009-z

**Published:** 2015-05-15

**Authors:** Julia Pederson, Rajiv B Kumar, Paula J Adams Hillard, Laura K Bachrach

**Affiliations:** Stanford University School of Medicine, Room H314, 300 Pasteur Drive, Stanford, CA USA; Department of Pediatrics, Stanford University School of Medicine, Stanford, CA USA; Department of Obstetrics and Gynecology, Stanford University School of Medicine, Stanford, CA USA

**Keywords:** Amenorrhea, Delayed puberty, Ovarian failure, Ovarian insufficiency

## Abstract

**Background:**

Primary ovarian insufficiency (POI) is characterized by 4 to 6 months of amenorrhea and elevated serum FSH and LH in females less than 40 years. Ovarian insufficiency is uncommon in pediatrics and typically results from a chromosomal abnormality or treatment for malignancy. Idiopathic POI in which no apparent precipitant is identified is even rarer. After encountering three teens with idiopathic POI in recent months, we utilized an informatics-enabled search of the electronic medical records from our hospital to identify all cases of idiopathic POI presenting from 1998–2013.

**Cases presentation:**

15 girls (ages 14.4 to 17.9 years) met criteria for idiopathic POI. At diagnosis, breast development ranged from Tanner stage 1 to 5; 6 of 15 patients had secondary amenorrhea. All patients presented in the past 11 years and 13 of 15 in the past 5 years.

**Conclusions:**

In this first case series of POI from the United States, we observed a clustering at our institution in recent years. If an increased incidence of idiopathic POI is identified at other institutions, further investigation into potential environmental and genetic precipitants is warranted.

## Background

Primary ovarian insufficiency (POI) is characterized by 4 to 6 months of amenorrhea, persistently elevated serum FSH and LH, and low serum estradiol in females younger than 40 years [1]. POI occurs in an estimated 1/10,000 women before the age of 30 years but is uncommon during adolescence [2]. When POI occurs in teens, it is typically iatrogenic (following oophorectomy or treatment for malignancy) or due to a chromosomal anomaly (Turner syndrome or Fragile X premutation) [1]. Case series of idiopathic POI in pediatrics have been reported from Italy [3] and Australia [4] involving 20 and 17 teens respectively between 1993 and 2006. To our knowledge, no series of idiopathic POI in adolescents has been reported in the United States.

We launched an informatics-enabled review of all medical records to determine the number and clinical presentation of teens with idiopathic POI. The review was approved by the Stanford University Institutional Review Board. The Stanford Translational Research Integrated Database Environment (STRIDE) patient cohort discovery tool provided us with the means to search all electronic medical records (EMR) between 1998 and 2013 [5]. Search criteria included age less than 19 years and ICD-9 diagnosis codes of 256.9 (unspecified ovarian dysfunction), 256.39 (other ovarian failure), 256.2 (post ablative ovarian failure) or 256.31 (premature menopause). Data extracted from the EMR included ICD-9 diagnoses, laboratory, pathology and radiology reports, clinical notes, pharmacy data, race, gender, zip code of residence and age at diagnoses.

## Cases presentation

The STRIDE search identified 136 patients; clinical notes were available for all but one. We reviewed these records to identify cases of idiopathic POI using the inclusion criteria of serum FSH and LH in the menopausal range, low or undetectable estradiol, normal 46,XX karyotype, and non-contributory past medical history. We excluded patients with a history of oophorectomy, chemotherapy, radiation or transplant [1].

The vast majority of patients identified had an iatrogenic cause (post malignancy, transplantation, or oophorectomy) or genetic mutation (Turner syndrome, fragile X permutation) leading to ovarian insufficiency (Table 1). Some had disorders not associated with ovarian insufficiency such as polycystic ovarian syndrome (PCOS), eating disorders or exercise associated amenorrhea, indicating an error in coding.

**Table 1:** **Diagnoses for 136 subjects from STRIDE search**

**Diagnosis**	**Number**	**% of total**
Idiopathic POI	15	11
Malignancy/Bone marrow transplant recipient	43	32
Turner syndrome	25	18
Organ transplant recipient	4	3
Galactosemia	2	1
Fragile X premutation	1	<1
Post-oophorectomy	4	3
Hypogonadotropic hypogonadism	27	20
CNS tumor = 8		
Idiopathic = 5		
Prader Willi syndrome = 1		
Delayed puberty = 7		
Exercise associated amenorrhea = 2		
Eating disorder = 3		
Depression = 1		
Other	14	10
Polycystic ovarian syndrome = 2		
Morbid obesity = 2		
Irregular menses = 4		
Wegener’s granulomatosis = 1		
20p12.1 deletion = 1		
46,XY males (CAH, androgen insensitivity, gonadal dysgenesis) = 4		
Incomplete clinical notes	1	<1

Fifteen females, ages 14.4 to 17.9 years, met our criteria for idiopathic POI (Table 2). All had repeated laboratory measurements showing elevated gonadotropins and a normal 46,XX karyotype. None had a history of illness, medication, or radiation to account for ovarian dysfunction. The 15 females presented between 2002 and 2013, and 13 of the 15 had been diagnosed since 2008. Heights, weights and BMI were largely normal. While 9 of the girls had primary amenorrhea, only 3 lacked all secondary sexual characteristics. Comorbidities included hypothyroidism (n = 1), depression with suicidal ideation (n = 2), IgA deficiency (n = 1), type 1 diabetes (n = 1) and tension headaches (n = 1). None of the patients reported a family history of ovarian insufficiency. No medications or environmental exposures were common to all. All females resided in Northern or Central California with no obvious zip code linkage.

**Table 2:** **Laboratory findings in POI cohort**

**Subject number**	**Year of diagnosis**	**Age (y)**	**Ethnicity** ^**a**^	**BMI (Z-score)**	**Height (Z-score)**	**Breast SMR** ^**b**^	**Type of amenorrhea**	**FSH (mIU/mL)** ^**c**^	**LH (mIU/mL)** ^**d**^	**Estradiol (pg/mL)** ^**e**^
1	2013	14.4	AI	−0.2	−2.6	1	Primary	60.0	12.3	<1.0
2	2013	15.1	NHW	−1.6	0.5	1	Primary	129.2	48.3	23.9
3	2013	16.4	NHW	1.4	−0.3	5	Primary	95.6	35.3	15.0
4	2012	15.1	AI	−0.3	0.1	5	Primary	18.7	34.3	8.0
5	2012	15.2	NHW	−1.7	−1.1	1	Primary	132.0	38.0	<1.0
6	2012	15.1	NHW	0.1	0.7	5	Primary	144.0	37.0	15.0
7	2012	15.2	Unknown	0	1.2	5	Secondary	74.6	28.5	<20
8	2011	17.2	Hispanic	1.2	−0.6	5	Secondary	77.9	31.4	<1.0
9	2010	16.4	Hispanic	1.1	0.9	5	Secondary	72.5	40.7	1.6
10	2009	17.1	Hispanic	0.8	−1.4	5	Secondary	106.0	51.0	18.0
11	2009	16.1	NHW	0.7	2.2	5	Secondary	48.0	23.0	<1.0
12	2008	15.3	AI	1.9	0.1	3	Primary	50.0	26.1	13.0
13	2008	17.9	AI	0.2	−0.5	5	Secondary	89.1	57.8	3.9
14	2006	17.1	Unknown	0.6	1.3	4	Primary	41.0	25.0	0.5
15	2002	16.1	Hispanic	0.5	−0.9	3	Primary	89.9	34.9	10.0

^a^AI = Asian -Indian. NHW = Non-Hispanic White. Unknown = not listed in medical record.

^b^Breast sexual maturity rating.

^c^Upper limit of normal reported as 12.8 mIU/mL.

^d^Upper limit of normal reported as 12 mIU/mL.

^e^Lower limit of detectable estradiol varied by commercial laboratory. Prepubertal normal range reported as 5–20 pg/mL.

Laboratory tests for autoimmunity were variably performed but negative in most subjects: none of 12 tested had adrenal antibodies and none of 7 had ovarian antibodies. Three of 10 patients tested had positive anti-thyroid peroxidase (TPO) antibodies but all 3 had normal thyroid function tests. None of 7 patients assessed had Fragile X premutations. None of 8 tested had detectable anti-Mullerian hormone.

Pelvic imaging by trans-abdominal ultrasound was performed on 10 females prior to estrogen therapy. This imaging failed to detect ovaries in 4 girls and a uterus in 2 of these 4. MRI was conducted for 3 of these 4 patients and also failed to identify the reportedly absent structures. Only one of these girls had Tanner 1 breasts. After starting estrogen replacement therapy, all of these patients had menstrual bleeding and were ultimately found to have pelvic structures (uterus or ovaries) on subsequent imaging.

## Conclusions

In this first case series of idiopathic POI from the United States, we identified 15 adolescents presenting since 1998 with 13 of 15 diagnosed in the past 5 years. Most patients in our cohort had partial or complete secondary sexual characteristics at presentation, and some had secondary amenorrhea. All warranted endocrine evaluation because of absence of breast development by age 13, lack of menses by age 15, or secondary amenorrhea [6].

The clinical presentation of idiopathic POI in these females was similar to that reported from Italy [4] and Australia [5]. However, we found no family history suggestive of genetic etiology. The Italian cohort included one set of affected monozygotic twins and a non-twin sister pair; another girl had a known family history of premature ovarian failure [3]. The Australian cohort included a girl with a family history of POI (4). We cannot fully exclude a genetic cause in our cohort, however, since we did not test for all genes linked to ovarian dysfunction on the X-chromosome and autosomes [7].

The majority of females in our cohort also lacked evidence of autoimmunity. One had type 1 diabetes and three had positive anti-TPO antibodies. Antibodies against the 21-hydroxylase enzyme and ovary were negative in all those who were tested. In the Italian series, 2 of the 20 girls had thyroid autoantibodies and 1 of these had clinical evidence of thyroiditis, celiac disease and rheumatoid arthritis [4]. One girl from the Australian series had positive thyroid antibodies and 7 of 17 had positive anti-nuclear antibodies [5]. Anti-ovarian and anti-adrenal antibodies were negative in all subjects in both the Italian and Australian series.

Transabdominal pelvic ultrasound prior to estrogen therapy failed to detect ovaries in 4 of 10 girls and uterus in 2; only one of these girls had Tanner 1 breasts. We suggest caution in concluding that a uterus or ovaries are absent in females with untreated POI, since ovaries are typically small if estrogen levels are prepubertal [8].

We recognize the limitations of these retrospective data from a single center. The EMR search was limited to the 15 year period for which STRIDE was available. Measurements of antibodies, fragile X analysis and pelvic imaging were not performed in all patients, as would be recommended for optimal evaluation. We could not definitively exclude changes in referral patterns as a contributing factor to the apparent increase in idiopathic POI cases in recent years. Adolescent Gynecology services were established only in 2007. However, all but two patients were initially referred by pediatricians to the Pediatric Endocrine division which has been seeing patients for more than three decades. The other two were referred by community endocrinologists. Because this was a retrospective chart review, we were limited in our ability to search for potential environmental exposures. We utilized zip codes as a rough proxy for this variable and found no clustering of cases.

Despite these limitations, we believe this case series of idiopathic POI has important clinical and research implications. Recognizing POI in teens who present with amenorrhea is essential in order to provide appropriate endocrine, gynecologic, and reproductive care and counseling [1,2]. If primary ovarian dysfunction is confirmed by repeated elevations in FSH and LH, a comprehensive work-up is indicated to determine the cause [1]. We will now be monitoring for idiopathic POI at our institution on a prospective basis to determine if more cases arise. In fact, two more teens have presented with this diagnosis since completion of the STRIDE search. We are hoping other institutions will determine if they are diagnosing idiopathic POI more frequently as well. If this is the case, a search for environmental precipitants and/or novel genetic causes is warranted.

## Consent

The study was approved by the Stanford University Institutional Review Board. No individual written consent was required for the STRIDE search. A copy of the waiver of consent is available to review by the Editor-in-Chief of this journal.

## Abbreviations

POI: Primary ovarian insufficiency
FSH: Follicle stimulating hormone
LH: Luteinizing hormone
STRIDE: Stanford translational research integrated database environment
MRI: Magnetic resonance imaging
PCOS: Polycystic ovarian syndrome
AI: Asian Indian
NHW: Non-Hispanic white
BMI: Body mass index
SMR: Sexual maturity rating
CNS: Central nervous system
